# The HOMO-LUMO Gap as Discriminator of Biotic from Abiotic Chemistries

**DOI:** 10.3390/life14101330

**Published:** 2024-10-18

**Authors:** Roman Abrosimov, Bernd Moosmann

**Affiliations:** 1 Evolutionary Biochemistry and Redox Medicine, Institute for Pathobiochemistry, University Medical Center of the Johannes Gutenberg University, 55128 Mainz, Germany; abrosrom@uni-mainz.de; 2 Institute for Quantitative and Computational Biosciences, Johannes Gutenberg University, 55128 Mainz, Germany

**Keywords:** abiogenesis, biosignature, extraterrestrial life, frontier orbital, life detection, molecular evolution, origin of life, secondary metabolism

## Abstract

Low-molecular-mass organic chemicals are widely discussed as potential indicators of life in extraterrestrial habitats. However, demarcation lines between biotic chemicals and abiotic chemicals have been difficult to define. Here, we have analyzed the potential utility of the quantum chemical property, HOMO-LUMO gap (HLG), as a novel proxy variable of life, since a significant trend towards incrementally smaller HLGs has been described in the genetically encoded amino acids. The HLG is a zeroth-order predictor of chemical reactivity. Comparing a set of 134 abiotic organic molecules recovered from meteorites, with 570 microbial and plant secondary metabolites thought to be exclusively biotic, we found that the average HLG of biotic molecules was significantly narrower (−10.4 ± 0.9 eV versus −12.4 ± 1.6 eV), with an effect size of g = 1.87. Limitation to hydrophilic molecules (XlogP < 2) improved the separation of biotic from abiotic compounds (g = 2.52). The “hydrophilic reactivity” quadrant defined by |HLG| < 11.25 eV and XlogP < 2 was populated exclusively by 183 biotic compounds and 6 abiotic compounds, 5 of which were nucleobases. We conclude that hydrophilic molecules with small HLGs represent valuable indicators of biotic activity, and we discuss the evolutionary plausibility of this inference.

## 1. Introduction

The detection of life on extraterrestrial planets will arguably depend on the identification of “biotic” chemicals whose eventual presence could only be explained by the activity of living systems. Typical examples of chemical compounds commonly considered biotic are the more recent and complex representatives of the proteinogenic amino acids [1,2,3] or informational polymers required for the chemical transmittance of information and, thus, biological inheritance [4,5,6]. Particular properties of organic chemicals such as homochirality have also been discussed as markers of biological activity [5,7]. The reliable discrimination of biotic chemicals (whose synthesis would require evolved catalytic activities typical of living systems) from abiotic chemicals (which would emerge in the absence of life through simple or modestly complex, plausible geochemical processes) is yet an unmet challenge in astrochemistry [8,9]. Among other reasons, this is due to the fact that various first-line building blocks of life, including amino acids as primordially relevant as cysteine [10,11], crucial sugars such as ribose [12], nucleobases [13,14] and nucleotides [15] have all been added to the list of abiotic chemicals in the last decade, weakening their intrinsic capacity to announce life.

The utilization of informational polymers as heralds of life is also burdened with difficulties, foremost of which is the arguably challenging verification that a specific, detected polymer was indeed informational. Regarding the “usual suspects” known from terrestrial life, peptides can also emerge from free amino acids or abiotic amino acid precursors in prebiotically plausible settings [10,16]. Similarly, nucleic acids can form from ribonucleotide triphosphates and other starting materials under abiotic conditions [15,17]. Eventually, polymer detection is intricate for various solubility [5] and stability [8,18] issues, especially since informational polymers will very likely arise from condensation reactions, such that degradation through pervasive hydrolysis has to be expected. In conclusion, additional new separators of biotic from abiotic chemistries are highly desirable, to aid in the detection of extraterrestrial life.

The HOMO-LUMO gap (HLG) is a quantum chemical property describing the energetic distance between two electronic energy levels, the Highest Occupied Molecular Orbital (HOMO) and the Lowest Unoccupied Molecular Orbital (LUMO) [19,20]. Both electronic energy levels are characteristic of a chemical structure and can readily be calculated for standard low-molecular-mass compounds with reasonable precision. Since many types of chemical reactions are kinetically (velocity-)controlled by the transfer of an electron from a HOMO to a LUMO, either within the same molecule or across two molecules, the HLG represents a versatile approximate predictor of chemical reactivity [20,21]. This applies foremost to photo-reactivity and redox reactivity [20,22,23]. The HLG stands in linear correlation with the historically earlier terms of chemical “hardness” (high charge density, low polarizability) and chemical “softness” (low charge density, high polarizability), the latter being equivalent to a small HLG [20].

In connection with the origin of life, the 20 standard amino acids of the genetic code displayed incrementally smaller HLGs when ordered by their presumed time of introduction into the genetic code [2,22]. In the same study, abiotic Murchison meteorite amino acids (n = 62) were sharply separated from a sample of modern redox cofactors (n = 15) by their much larger HLGs [22]. Inspired by this observation and by the proposal and prototype design of a future life-detection instrument whose measuring principle relates to the HLG [24,25], we have here investigated an expanded set of reference compounds (n = 704) to explore the potential suitability of the HLG as a general discriminator of biotic from abiotic chemistries. Secondary metabolites were chosen as biotic reference group for their reliably biochemical (rather than geochemical) origin, whereas organic compounds detected in carbonaceous meteorites were adopted as abiotic reference group.

## 2. Materials and Methods

### 2.1. Sampling of Compounds

Carbonaceous meteorites such as the Murchison meteorite can contain numerous organic compounds of biological interest, including amino acids and nucleobases [14,26]. Beyond these well-characterized components, the Murchison meteorite may host tens of thousands to millions of other low-molecular-weight organic chemicals at, necessarily, low concentrations [27,28]. To apply an implicit threshold that would prefer more highly concentrated compounds, abiotic meteorite organic chemicals were arbitrarily collected from the classical literature rather than from recent high-end technology reports. Specifically, nine authoritative references were consulted to assemble a tentatively representative list of 134 meteorite organic compounds detected in the Murchison meteorite [29,30,31,32,33], Murchison and Murray meteorite [34,35], Murchison and Yamato-791198 meteorite [36], and Murchison, Bells, and Ivuna meteorite [37].

A variety of 570 plant or microbial secondary metabolites were compiled from the Kyoto Encyclopedia of Genes and Genomes (KEGG) [38] as biotic reference group. A minimum of 30 representatives were selected from the KEGG for each of the following seven classes of secondary metabolites: flavonoids (102), lysine alkaloids (75), ornithine alkaloids (99), phenylpropanoids (48), polyketides (42), shikimate derivatives (31), and terpenoids (173). A compound list is provided in the Supplementary Material (Data S1).

### 2.2. Determination of Compound Properties

All chemical structures and their XlogP values were obtained from NCBI PubChem [39]. The XlogP value is a dimensionless index of hydrophobicity and denotes the decadic logarithm of the octanol/water partition coefficient of a certain compound. Chemical structures were retrieved as isomeric SMILES (Simplified Molecular Input Line Entry System) codes, which represent a chemical’s three-dimensional structure in the form of a linear string of symbols. Isomeric SMILES codes of the investigated compounds were transferred to the commercial ChemBioUltra 13.0 software package (PerkinElmer, Waltham, MA, USA) and processed as described [22]. In brief, molecular mechanics geometry optimization adopting the MM2 force field algorithm of ChemBioUltra was performed before quantum chemical energy minimization and orbital energy determination. These ab initio calculations were accomplished with the Restricted Hartree–Fock (RHF) algorithm implemented in GAMESS-US [40,41] and executed via the GAMESS-US interface of ChemBioUltra. The adopted RHF basis set was 6-31+G(d), thus including diffuse and polarization functions on heavy atoms. HLGs were subsequently calculated from the identified frontier orbital energy levels.

### 2.3. Data Evaluation and Statistics

Data were described and visualized using SigmaPlot 11.0 (Systat, Richmond, CA, USA). Linear regressions were performed with the same software. Parametric and nonparametric Analyses of Variance (ANOVA) were conducted with SPSS 23 (IBM, Armonk, NY, USA). Effect sizes according to Hedges and Cohen were calculated with an online tool [42].

## 3. Results

Secondary metabolites are highly individual compounds that belong to a finite number of families designated by their founding precursor, such as the lysine alkaloid family. From this primary metabolite precursor, they are biosynthesized in usually lengthy, multi-step, linear metabolic pathways [43,44]. Secondary metabolites are generally non-essential for their producer under optimal conditions but often provide a significant survival or growth advantage under suboptimal conditions like drought, irradiation, and nutrient shortage, or in the presence of biological competitors or predators [45,46]. The multi-step biosynthesis of the secondary metabolites as well as their non-universality and phylogenetic restriction indicate that their origin is exclusively biotic; there was no overlap between any of the secondary metabolites investigated in this study and any reported meteorite compound. In contrast, various primary metabolites of life such as amino acids, carbohydrates, and nucleobases were contained in the abiotic meteorite compound group.

**Figure 1:**
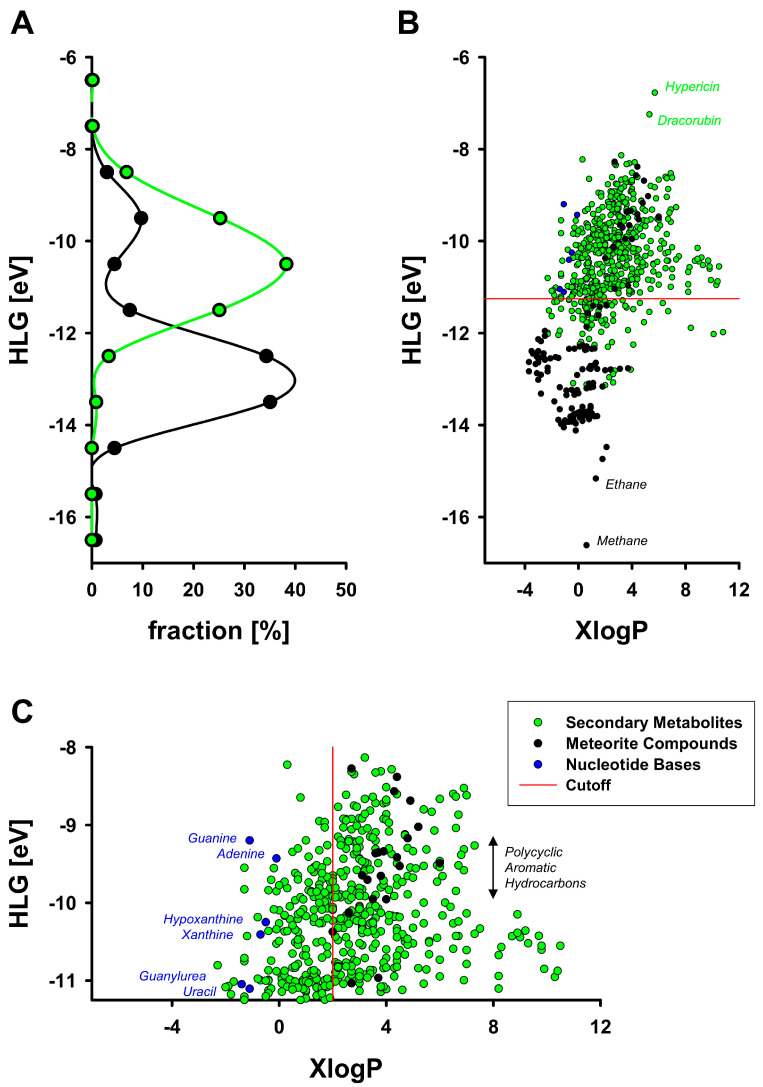
The HOMO-LUMO gap (HLG) in secondary metabolites and meteorite organic compounds. (**A**) Normalized distribution of the HLGs in 570 secondary metabolites (green) and 134 meteorite compounds (black). Data points indicate the percentage (“fraction”) of compounds encountered in the ±0.5 eV HLG interval surrounding the data point. (**B**) Scatter plot of the HLGs of all investigated compounds versus their hydrophobicity (XlogP value). Selected compounds are named. Five nucleobases and guanylurea in the meteorite group are marked in blue. The horizontal demarcation line at y = −11.25 eV (red) separates the secondary metabolites (green) by 480/90 (top/bottom) and the meteorite compounds (black and blue) by 27/107 (top/bottom). (**C**) Magnification of the high-reactivity compounds (|HLG| < 11.25 eV). Secondary metabolites (478) are shown in green; meteorite compounds (27) are shown in black (21) or blue (6). The black compounds mostly represent polycyclic aromatic hydrocarbons (PAHs), whereas the blue compounds represent nucleobases and guanylurea, as indicated. The vertical demarcation line at XlogP = 2 (red) separates the secondary metabolites (green) by 183/297 (left/right) and the meteorite compounds (black and blue) by 6/21. There are no black compounds left of this cutoff line. Except for nucleobases, the combination of high chemical reactivity (a narrow HLG) with hydrophilicity (a small XlogP) is characteristic of biotic secondary metabolites. HLGs are given in electronvolt (eV; 1 eV = 96.5 kJ/mol); XlogP values are dimensionless.

Analysis of the distribution of the HOMO-LUMO gaps (HLGs) across the abiotic meteorite compounds and the biotic secondary metabolites demonstrated that the biotic metabolites had substantially smaller HLGs (Figure 1A), on average by 2 eV (corresponding to ~200 kJ/mol). For comparison, the hydrolysis of ATP to ADP yields about 0.3 eV. The separation of the two groups of compounds was statistically highly significant and characterized by effect sizes of 1.6–1.9, depending on the method (Table 1). Still, baseline separation of the secondary metabolites from the meteorite compounds was not achieved. Because most of the meteorite compounds with small HLGs appeared to be water-insoluble polycyclic aromatic hydrocarbons (PAHs), hydrophobicity was introduced as a second parameter to potentially improve the separation of biotic from abiotic compounds. To this end, calculated octanol/water partition coefficients (XlogP values; a commonly used index of hydrophobicity) were assigned to each compound, and HLGs were plotted against these XlogP values thereafter (Figure 1B).

Altogether, secondary metabolites were more hydrophobic than meteorite compounds by approximately two orders of magnitude, and, in both groups of compounds, increased chemical reactivity in terms of a narrower HLG was correlated with increased hydrophobicity (Table 1). Since the majority of the meteorite compounds exhibited HLGs wider than 11.25 eV, this value was selected as a bona fide delimiter between meteoritic and metabolic chemicals (Figure 1B). Inspecting the compounds above this threshold more closely (Figure 1C), a preponderance of secondary metabolites was evident, which was yet interrupted by two smaller clusters of meteorite compounds running through the metabolites in a quasi-linear, parallel fashion: the more hydrophobic cluster mostly consisted of PAHs, whereas the more hydrophilic cluster happened to be composed of five nucleobases and the related linear molecule guanylurea. Guanylurea was originally reported to occur in the Orgueil meteorite at a very high concentration of 270 ppm, together with the purines adenine and guanine at approximately 10-fold lower concentrations [47]. Modern equipment has confirmed the presence of purine and pyrimidine bases in various carbonaceous chondrites, but at substantially lower concentrations in the ppb range [13,14].

Application of a second delimiter at XlogP = 2 to separate the chemically more reactive compounds in Figure 1C into a hydrophilic and a hydrophobic subset generated a quadrant of concomitantly reactive and hydrophilic chemicals that was populated exclusively by 183 biotic compounds, five nucleobases, and guanylurea. Hence, the HLG as a proxy variable of biotic activity appears to be most expedient when applied after selection for compound hydrophilicity. This inference was also supported statistically, as restriction to hydrophilic compounds with XlogP < 2 increased the quality of the separation of secondary metabolites from meteorite compounds (Table 1). More specifically, the mean HLG difference between the two groups increased to 2.3 eV, the *p* values remained stable despite a smaller number of compounds, and the effect sizes rose to 2.4–2.5.

## 4. Discussion

In analyzing 134 abiotic organic chemicals that have been evidenced to occur in meteorites and 570 secondary metabolites biosynthesized by terrestrial plants and microbes, we find that both groups of compounds are well distinguished by their quantum chemical HOMO-LUMO gaps (HLGs), which were smaller by 2 eV on average in the secondary metabolites (Table 1). Considering that no preselection was applied during the arbitrary assembly of the compound arrays beyond the statutory criteria of “meteorite occurrence” and literature classification as “secondary metabolite”, respectively, the 2 eV difference is intriguing. As visible from Figure 1B, the chemical space of organic chemistry HLGs spans approximately 10 eV, from the simplest molecules like methane and ethane to very complex and already unstable molecules like dracorubin and hypericin. Omitting these four outliers, almost all compounds fall within a 6 eV window ranging from −14 eV to −8 eV. Comparison of the HLGs of chemically inert amino acids from the Murchison meteorite with modern redox cofactors derived from shikimate has demonstrated an approximate HLG difference of 3 eV between these oppositional compound sets [22]. In pharmacological analyses of cytoprotective chain-breaking antioxidants, differences of about 1 eV in the energetic level of the SOMO (the Singly Occupied Molecular Orbital, a variant of the HOMO encountered in radicals) explained the disparity between a modest and an excellent antioxidant [48,49]. In photosynthetic reaction centers, differences in the HLGs of the contained (bacterio)chlorophylls and (bacterio)pheophytins in the order of 0.2 eV accounted for the tuned photoexcitation properties of these chomophores, which have functional consequences [23]. In summary, an HLG difference of 2 eV on average between two acceptably large compound arrays of defined, disparate origin is sufficient to imply biological relevance.

Several aspects about secondary metabolites as potential proxies of life are of interest from a theoretical as well as a practical point of view. When detected in an extraterrestrial setting, secondary metabolite structures would imply biotic activity with considerable confidence, because, on Earth, they generally emanate from multi-step metabolic pathways. For example, quercetin, the prototypical high-concentration flavonoid from onions and other plants, requires 16 committed steps to be synthesized from 3-deoxy-D-arabino-heptulosonic acid 7-phosphate (DAHP) [38]. The exclusively biotic origin of terrestrial secondary metabolites is also implied by the individuality and usually restricted usage of these compounds by specific phylogenetic branches of life. Were some of these compounds easily available from abiotic sources, one might expect a more widespread deployment.

As secondary metabolites often represent toxins to combat rivaling life forms or provide other specific advantages in competitive, multi-species biotic communities [45,46,50,51,52,53], they may be strong indicators of ongoing Darwinian processes on other planets. Concomitantly, they may be less suitable for the detection of clonal, single-species lineages of life, if those should exist, because such lineages would not be anticipated to produce many secondary metabolites. The “Darwinian indicator” feature of the secondary metabolites thus reduces the overall danger of false-positive signals when using these molecules as proxies of life. On the other hand, their individuality and restriction to specific phylogenetic branches also reduce the probability of finding a true-positive signal; in the end, secondary metabolites are not essential to life.

Despite this circumstance, secondary metabolites on Earth are widely encountered even in extreme habitats that would be regarded as particularly relevant to the search for extraterrestrial life. For example, secondary metabolites are very common in thermophilic and psychrophilic as well as halophilic microbial communities [54]. They have been confirmed in thermophilic bacterial strains from microbial mats in the Atacama Desert at high altitudes [55], in deep-sea cold seeps where no photosynthesis occurs [56], and in acid mine drainage—an acidic, metal-enriched, corrosive habitat generated by human mining activity [57]. Therefore, within terrestrial limits, the heightened reactivity of the secondary metabolites does not seem to hamper their biological utility at extreme temperatures or pH values. Quite the contrary, microbes under strong eco-evolutionary pressure may actually produce a more diverse set of secondary metabolites than unstressed species [54,55,56].

The statistical detection disadvantage that comes with the individuality of the secondary metabolites may also be compensated in part by the fact that when they do occur, they often occur at high concentrations, making them rather easily detectable [43]. For instance, the flavonoid quercetin is synthesized to an amount of 3–12% of the total dry weight in onions [53]. The alkaloid nicotine is present at 0.5–8% by weight in dry tobacco leaves [58]. At least in plants, there is, furthermore, no indication that investment into a single secondary metabolite negatively affects the production of other such metabolites, since multiple secondary metabolites often exist in combination [50]. Clearly, abiotic polycyclic aromatic hydrocarbons (PAHs) with their narrow HLGs can also be highly concentrated in meteorites [59]. Therefore, it will be important to consider additional molecular indicators such as hydrophilicity when consulting the HLG as a proxy variable of life. For instance, collateral solubility features such as differential water solubility at specific pH values (as seen in alkaloids) might be used to refine the separation of biotic secondary metabolites from otherwise false-positively detected PAHs.

Epistemologically, the definitive presence of PAHs with very small HLGs in meteorites is relevant since it provides direct evidence that a narrow HLG is not prohibitory of space travel and long half-lives in meteorites. Therefore, the underrepresentation of reactive and concomitantly water-soluble compounds in meteorites, as seen here, may indeed reflect a lack of abiotic geochemical activity towards their synthesis, and not just their later, selective decomposition. This conclusion is supported by laboratory experiments showing the facile photo-destruction of Murchison extracts, indicating that little destructive activity must have been present during space travel [27]. Other extraterrestrial sources of organic chemicals such as the Moon [60] and Mars [61], and returned material from asteroid sampling missions [62] should be analyzed to corroborate this conclusion and to refine the HLG model of biotic–abiotic discrimination.

## 5. Conclusions

Biotic and abiotic chemicals can be statistically distinguished at the quantum chemical level by the substantially smaller HLGs of the former compounds. This observation stands to reason because life may be incompletely viewed as an assembly of compounds under selection for heightened chemical reactivity in an aqueous medium [2,22]. The evolutionary demand for heightened aqueous reactivity is not per se trivial to fulfill, since the general chemical trend points towards increased lipophilicity as a statistical prerequisite for a small HLG (Figure 1B, Table 1). Strikingly, the visible outliers among the meteorite compounds that already combined hydrophilicity with reactivity in a “para-biotic” fashion were nucleobases and, thus, essential precursors to life as encountered on Earth. Our results support recent advances to develop life-detection instruments based on the measurement of single-molecule quantum electronic tunneling, a detection method related to the width of the HLG [24,25].

## Figures and Tables

**Table 1:** Statistical analysis of HOMO-LUMO gap (HLG) size and hydrophobicity (XlogP value) in secondary metabolites and meteorite organic compounds.

	Secondary Metabolites (All)	Meteorite Compounds (All)	Secondary Metabolites (Hydrophilic: XlogP < 2)	Meteorite Compounds (Hydrophilic: XlogP < 2)
Number of compounds	570	134	233	107
Mean HLG [eV] (± SD)	−10.4 ± 0.9	−12.4 ± 1.6	−10.7 ± 0.8	−13.0 ± 1.1
Mean XlogP (± SD)	2.6 ± 2.3	0.2 ± 2.2	0.6 ± 1.0	−0.7 ± 1.5
Correlation HLG vs. XlogP	r = 0.259; *p* = 4 × 10^−10^	r = 0.501; *p* = 7 × 10^−10^	r = 0.254; *p* = 9 × 10^−5^	r = −0.203; *p* = 4 × 10^−2^
ANOVA	F = 380; df = 703; *p* = 6 × 10^−68^		F = 465; df = 339; *p* = 2 × 10^−65^	
ANOVA on ranks	U = 11,700; n = 704; *p* = 8 × 10^−36^		U = 1210; n = 340; *p* = 9 × 10^−41^	
Effect size (Hedges)	g = 1.87		g = 2.52	
Effect size (Cohen)	d = 1.56		d = 2.39	

## Data Availability

The data supporting this article are included as Supplementary Material.

## Supplementary Materials

The following supporting information can be downloaded at https://www.mdpi.com/article/10.3390/life14101330/s1, Data S1: List of compounds and compound properties.

## References

[1] Longo L.M., Blaber M. (2012). Protein design at the interface of the pre-biotic and biotic worlds. Arch. Biochem. Biophys..

[2] Moosmann B. (2021). Redox biochemistry of the genetic code. Trends Biochem. Sci..

[3] Mayer-Bacon C., Meringer M., Havel R., Aponte J.C., Freeland S. (2022). A closer look at non-random patterns within chemistry space for a smaller, earlier amino acid alphabet. J. Mol. Evol..

[4] Pinheiro V.B., Taylor A.I., Cozens C., Abramov M., Renders M., Zhang S., Chaput J.C., Wengel J., Peak-Chew S.-Y., McLaughlin S.H. (2012). Synthetic genetic polymers capable of heredity and evolution. Science.

[5] Benner S.A. (2017). Detecting Darwinism from molecules in the Enceladus plumes, Jupiter’s moons, and other planetary water lagoons. Astrobiology.

[6] McKaig J.M., Kim M.G., Carr C.E. (2023). Translation as a biosignature. BioRxiv.

[7] Glavin D.P., Burton A.S., Elsila J.E., Aponte J.C., Dworkin J.P. (2020). The search for chiral asymmetry as a potential biosignature in our solar system. Chem. Rev..

[8] Neveu M., Hays L.E., Voytek M.A., New M.H., Schulte M.D. (2018). The ladder of life detection. Astrobiology.

[9] Barge L.M., Rodriguez L.E., Weber J.M., Theiling B.P. (2022). Determining the “Biosignature Threshold” for life detection on biotic, abiotic, or prebiotic worlds. Astrobiology.

[10] Foden C.S., Islam S., Fernández-García C., Maugeri L., Sheppard T.D., Powner M.W. (2020). Prebiotic synthesis of cysteine peptides that catalyze peptide ligation in neutral water. Science.

[11] Moosmann B., Schindeldecker M., Hajieva P. (2020). Cysteine, glutathione and a new genetic code: Biochemical adaptations of the primordial cells that spread into open water and survived biospheric oxygenation. Biol. Chem..

[12] Furukawa Y., Chikaraishi Y., Ohkouchi N., Ogawa N.O., Glavin D.P., Dworkin J.P., Abe C., Nakamura T. (2019). Extraterrestrial ribose and other sugars in primitive meteorites. Proc. Natl. Acad. Sci. USA.

[13] Callahan M.P., Smith K.E., Cleaves H.J., Ruzicka J., Stern J.C., Glavin D.P., House C.H., Dworkin J.P. (2011). Carbonaceous meteorites contain a wide range of extraterrestrial nucleobases. Proc. Natl. Acad. Sci. USA.

[14] Oba Y., Takano Y., Furukawa Y., Koga T., Glavin D.P., Dworkin J.P., Naraoka H. (2022). Identifying the wide diversity of extraterrestrial purine and pyrimidine nucleobases in carbonaceous meteorites. Nat. Commun..

[15] Yadav M., Kumar R., Krishnamurthy R. (2020). Chemistry of abiotic nucleotide synthesis. Chem. Rev..

[16] Holden D.T., Morato N.M., Cooks R.G. (2022). Aqueous microdroplets enable abiotic synthesis and chain extension of unique peptide isomers from free amino acids. Proc. Natl. Acad. Sci. USA.

[17] Jerome C.A., Kim H.J., Mojzsis S.J., Benner S.A., Biondi E. (2022). Catalytic synthesis of polyribonucleic acid on prebiotic rock glasses. Astrobiology.

[18] Saladino R., Crestini C., Ciciriello F., Di Mauro E., Costanzo G. (2006). Origin of informational polymers: Differential stability of phosphoester bonds in ribomonomers and ribooligomers. J. Biol. Chem..

[19] Fukui K. (1982). Role of frontier orbitals in chemical reactions. Science.

[20] Pearson R.G. (1986). Absolute electronegativity and hardness correlated with molecular orbital theory. Proc. Natl. Acad. Sci. USA.

[21] Arnett E.M., Ludwig R.T. (1995). On the relevance of the Parr-Pearson principle of absolute hardness to organic chemistry. J. Am. Chem. Soc..

[22] Granold M., Hajieva P., Toşa M.I., Irimie F.D., Moosmann B. (2018). Modern diversification of the amino acid repertoire driven by oxygen. Proc. Natl. Acad. Sci. USA.

[23] Tamura H., Saito K., Ishikita H. (2020). Acquirement of water-splitting ability and alteration of the charge-separation mechanism in photosynthetic reaction centers. Proc. Natl. Acad. Sci. USA.

[24] Carr C.E., Ramírez-Colón J.L., Duzdevich D., Lee S., Taniguchi M., Ohshiro T., Komoto Y., Soderblom J.M., Zuber M.T. (2023). Solid-state single-molecule sensing with the Electronic Life-Detection Instrument for Enceladus/Europa (ELIE). Astrobiology.

[25] Ramírez-Colón J.L., Johnson E., Duzdevich D., Lee S., Soderblom J., Zuber M.T., Taniguchi M., Ohshiro T., Komoto Y., Carr C.E. (2024). Nanogap solid-state single-molecule detection at Mars, Europa, and microgravity conditions. BioRxiv.

[26] Glavin D.P., McLain H.L., Dworkin J.P., Parker E.T., Elsila J.E., Aponte J.C., Simkus D.N., Pozarycki C.I., Graham H.V., Nittler L.R. (2020). Abundant extraterrestrial amino acids in the primitive CM carbonaceous chondrite Asuka 12236. Meteorit. Planet. Sci..

[27] Orthous-Daunay F.-R., Piani L., Flandinet L., Thissen R., Wolters C., Vuitton V., Poch O., Moynier F., Sugawara I., Naraoka H. (2019). Ultraviolet-photon fingerprints on chondritic large organic molecules. Geochem. J..

[28] Schmitt-Kopplin P., Gabelica Z., Gougeon R.D., Fekete A., Kanawati B., Harir M., Gebefuegi I., Eckel G., Hertkorn N. (2010). High molecular diversity of extraterrestrial organic matter in Murchison meteorite revealed 40 years after its fall. Proc. Natl. Acad. Sci. USA.

[29] Jungclaus G.A., Cronin J.R., Moore C.B., Yuen G.U. (1976). Aliphatic amines in the Murchison meteorite. Nature.

[30] Cooper G.W., Onwo W.M., Cronin J.R. (1992). Alkyl phosphonic acids and sulfonic acids in the Murchison meteorite. Geochim. Cosmochim. Acta.

[31] Cooper G.W., Cronin J.R. (1995). Linear and cyclic aliphatic carboxamides of the Murchison meteorite: Hydrolyzable derivatives of amino acids and other carboxylic acids. Geochim. Cosmochim. Acta.

[32] Sephton M.A. (2002). Organic compounds in carbonaceous meteorites. Nat. Prod. Rep..

[33] Lerner N., Cooper G. (2005). Iminodicarboxylic acids in the Murchison meteorite: Evidence of Strecker reactions. Geochim. Cosmochim. Acta.

[34] Cooper G., Kimmich N., Belisle W., Sarinana J., Brabham K., Garrel L. (2001). Carbonaceous meteorites as a source of sugar-related organic compounds for the early Earth. Nature.

[35] Pizzarello S., Cooper G.W., Flynn G.J., McSween H.Y., Lauretta D.S. (2006). The Nature and Distribution of the Organic Material in Carbonaceous Chondrites and Interplanetary Dust Particles. Meteorites and the Early Solar System II.

[36] Shimoyama A., Shigematsu R. (1994). Dicarboxylic acids in the Murchison and Yamato-791198 carbonaceous chondrites. Chem. Lett..

[37] Monroe A.A., Pizzarello S. (2011). The soluble organic compounds of the Bells meteorite: Not a unique or unusual composition. Geochim. Cosmochim. Acta.

[38] Kyoto Encyclopedia of Genes and Genomes (KEGG). www.genome.jp/kegg.

[39] National Center for Biotechnology Information PubChem. www.pubchem.ncbi.nlm.nih.gov.

[40] Schmidt M.W., Baldridge K.K., Boatz J.A., Elbert S.T., Gordon M.S., Jensen J.H., Koseki S., Matsunaga N., Nguyen K.A., Su S. (1993). General atomic and molecular electronic structure system. J. Comput. Chem..

[41] General Atomic and Molecular Electronic Structure System—US Version (GAMESS-US). www.msg.chem.iastate.edu.

[42] Social Science Statistics. www.socscistatistics.com.

[43] Wink M., Wink M. (2010). Introduction: Biochemistry, Physiology and Ecological Functions of Secondary Metabolites. Annual Plant Reviews Volume 40: Biochemistry of Plant Secondary Metabolism.

[44] Weng J.K., Philippe R.N., Noel J.P. (2012). The rise of chemodiversity in plants. Science.

[45] Erb M., Kliebenstein D.J. (2020). Plant secondary metabolites as defenses, regulators, and primary metabolites: The blurred functional trichotomy. Plant Physiol..

[46] Divekar P.A., Narayana S., Divekar B.A., Kumar R., Gadratagi B.G., Ray A., Singh A.K., Rani V., Singh V., Singh A.K. (2022). Plant secondary metabolites as defense tools against herbivores for sustainable crop protection. Int. J. Mol. Sci..

[47] Hayatsu R., Studier M.H., Oda A., Fuse K., Anders E. (1968). Origin of organic matter in early solar system—II. *Nitrogen compounds*. Geochim. Cosmochim. Acta.

[48] Moosmann B., Skutella T., Beyer K., Behl C. (2001). Protective activity of aromatic amines and imines against oxidative nerve cell death. Biol. Chem..

[49] Ohlow M.J., Granold M., Schreckenberger M., Moosmann B. (2012). Is the chromanol head group of vitamin E nature’s final truth on chain-breaking antioxidants?. FEBS Lett..

[50] Koricheva J., Nykänen H., Gianoli E. (2004). Meta-analysis of trade-offs among plant antiherbivore defenses: Are plants jacks-of-all-trades, masters of all?. Am. Nat..

[51] Kroymann J. (2011). Natural diversity and adaptation in plant secondary metabolism. Curr. Opin. Plant Biol..

[52] Alam K., Mazumder A., Sikdar S., Zhao Y.-M., Hao J., Song C., Wang Y., Sarkar R., Islam S., Zhang Y. (2022). Streptomyces: The biofactory of secondary metabolites. Front. Microbiol..

[53] Kwak J.H., Seo J.M., Kim N.H., Arasu M.V., Kim S., Yoon M.K., Kim S.J. (2017). Variation of quercetin glycoside derivatives in three onion (*Allium cepa* L.) varieties. Saudi, J. Biol. Sci..

[54] Hatti-Kaul R., Abouhmad A., Lee N. (2020). Extremophiles: A Promising Source of Novel Natural Products. Biotechnological Applications of Extremophilic Microorganisms.

[55] Pardo-Esté C., Cortés J., Castro-Severyn J., Pérez V., Henriquez-Aedo K., Cuadros F., Yañez C., Cuadros-Orellana S., Dorador C., Molina V. (2024). Secondary metabolites with antimicrobial activity produced by thermophilic bacteria from a high-altitude hydrothermal system. Front. Microbiol..

[56] Dong X., Zhang T., Wu W., Peng Y., Liu X., Han Y., Chen X., Gao Z., Xia J., Shao Z. (2024). A vast repertoire of secondary metabolites potentially influences community dynamics and biogeochemical processes in cold seeps. Sci. Adv..

[57] Wang L., Liu W., Liang J., Zhao L., Li Q., Zhou C., Cen H., Weng Q., Zhang G. (2022). Mining of novel secondary metabolite biosynthetic gene clusters from acid mine drainage. Sci. Data.

[58] Henry J.B., Vann M.C., Lewis R.S. (2019). Agronomic practices affecting nicotine concentration in flue-cured tobacco: A review. Agron. J..

[59] Lecasble M., Remusat L., Viennet J.C., Laurent B., Bernard S. (2022). Polycyclic aromatic hydrocarbons in carbonaceous chondrites can be used as tracers of both pre-accretion and secondary processes. Geochim. Cosmochim. Acta.

[60] Elsila J.E., Aponte J.C., McLain H.L., Simkus D.N., Dworkin J.P., Glavin D.P., Zeigler R.A., McCubbin F.M., The ANGSA Science Team (2024). Soluble organic compounds and cyanide in Apollo 17 lunar samples: Origins and curation effects. J. Geophys. Res. Planets.

[61] Ansari A.H. (2023). Detection of organic matter on Mars, results from various Mars missions, challenges, and future strategy: A review. Front. Astron. Space Sci..

[62] Parker E.T., Chan Q.H., Glavin D.P., Dworkin J.P. (2022). Non-protein amino acids identified in carbon-rich Hayabusa particles. Meteorit. Planet. Sci..

