# Supplementary material for: The HOMO-LUMO Gap as Discriminator of Biotic from Abiotic Chemistries
Source: Life (Basel). 2024 Oct 18;14(10):1330. doi: 10.3390/life14101330 (PMC11509606; doi:10.3390/life14101330)
Supplement: Supplementary file 1 [file life-14-01330-s001.zip › life-3260636-supplementary.pdf]

Data S1. List of compounds and compound properties.

Meteorite compounds	HLG [eV]	XLogP
1-Methylethanesulfonic acid	-13.24	-0.1
1-Phenylnaphtalene	-9.17	4.8
2-Butanone	-12.79	0.3
2-Decanone	-12.77	3.7
2-Heptanone	-12.81	2.0
2-Hexanone	-12.78	1.4
2-Hydroxyisobutyric acid	-13.83	-0.4
2-Methylbutyralaldehyde	-13.09	1.1
2-Nonanone	-12.78	3.1
2-Oktanone	-12.79	2.4
2-Pentanone	-12.78	0.9
3-Hexanone	-12.69	1.2
3-Methyl-2-butanone	-12.77	0.8
3-Methyl-2-pentanone	-12.65	1.3
3-Pentanone	-12.72	0.9
9H-Carbazole	-9.35	3.7
α-Aminobutyric acid	-12.58	-2.5
α-Aminoisobutyric acid	-12.91	-2.8
Acenaphthene	-9.34	3.9
Acetaldehyde	-13.34	-0.3
Acetic acid	-14.12	-0.2
Acetone	-12.91	-0.1
Acetophenone	-11.43	1.6
Adenine	-9.43	-0.1
Adipic acid	-13.72	0.1
Anthracen-9(10H)-one	-10.97	3.7
Anthracene	-8.38	4.4
Anthracenedione	-8.28	2.7
Benzaldehyde	-11.61	1.5
Benzanthracen-7-one	-8.57	4.3
Benzene	-11.38	2.1
Benzothiazole	-10.37	2.0
Benzothiophene	-9.65	3.1
Biphenyldicarbonitrile	-9.65	3.8
Biphenyl	-9.96	4.0
Butylamine	-12.34	1.0
Butyralaldehyde	-13.18	0.9
Butyric acid	-13.87	0.8
Cycloleucine	-12.53	-2.6
D-Alanine	-12.64	-3.0
D-Glutamic acid	-12.83	-3.7
Dibenzothiophene	-9.42	4.4
Diethylamine	-11.86	0.6
Dihydroxyacetone	-13.18	-1.4
L-2-Hydroxy-2-methyl butyric acid	-13.73	0.3
L-2-Hydroxy-2-methyl valeric acid	-13.71	0.6
L-2-Hydroxy-3-methyl butyric acid	-13.76	-0.3
D-2-Hydroxy-3-methyl valeric acid	-13.59	0.9
L-2-Hydroxybutyric acid	-13.84	0.1
L-2-Hydroxycaproic acid	-13.75	1.0
L-2-Hydroxyglutaric acid	-13.90	-1.0
L-2-Hydroxyisocarpoic acid	-13.63	0.9
L-2-Hydroxyvaleric acid	-13.80	0.4
L-3-Hydroxybutyric acid	-13.64	-0.5
L-3-Hydroxyisobutyric acid	-13.80	-0.4
D-Citramalic acid	-13.93	-0.9
L-Lactic acid	-13.93	-0.7
L-Malic acid	-13.98	-1.3

Ethanesulfonic acid	-13.23	-0.5
Ethanol	-13.74	-0.1
Ethene	-12.34	1.2
Ethylamine	-12.34	-0.3
Ethylene glycol	-13.66	-1.4
Fluoranthene	-9.02	5.2
Fluoren-9-one	-9.36	3.6
Formaldehyde	-13.81	1.2
Glutaric acid	-13.93	-0.3
Glyceric acid	-13.89	-1.5
Glycerol	-13.49	-1.8
Glycine	-12.67	-3.2
Glycolaldehyde	-13.35	-0.9
Glycolic acid	-14.05	-1.1
Guanine	-9.20	-1.1
Guanylurea	-11.05	-1.4
Hexanal	-13.16	1.8
Homophthalimide	-11.56	0.7
Hypoxanthine	-10.25	-0.5
Iminodiacetic acid	-12.44	-3.3
Iminodipropionic acid	-12.39	-3.5
Iminopropionicacetic acid	-12.45	-3.4
Isobutyraldehyde	-13.10	0.8
Isobutyric acid	-13.83	0.8
Isopropanol	-13.61	0.3
Isopropylamine	-12.29	0.1
Isovaline	-12.83	-2.3
L-Alanine	-13.02	-3.0
L-Aspartic acid	-13.32	-2.8
L-Glutamic acid	-12.63	-3.7
L-Isoleucine	-12.52	-1.7
L-Leucine	-12.54	-1.5
L-Proline	-11.95	-2.5
L-Serine	-12.87	-3.1
L-Valine	-12.75	-2.3
Methanesulfonic acid	-13.25	-0.9
Methanol	-13.93	-0.5
Methylamine	-12.34	-0.7
Methylsuccinic acid	-13.96	-0.2
Naphtalene	-9.70	3.3
N-Methyl alanine	-12.06	-2.5
N-Methyl β-alanine	-12.14	-3.0
Norvaline	-12.55	-2.1
o-Terphenyl	-9.49	6.0
Phenanthrene	-9.53	4.5
Phenanthridine	-9.95	3.5
Phenyl pyridine	-10.14	2.6
Phthalimide	-11.41	1.1
Propanesulfonic acid	-13.31	0.0
Propionaldehyde	-13.20	0.6
Proprionic acid	-13.91	0.3
Propylamine	-12.37	0.5
Pyrene	-8.69	4.9
Sarcosine	-12.18	-2.8
sec-Butylamine	-12.29	0.6
Succinic acid	-13.82	-0.6
tert-Butylamine	-12.27	0.3
Toluene	-11.04	2.7
Uracil	-11.11	-1.1
Valeraldehyde	-13.17	1.1
Valeric acid	-13.81	1.4
Xanthine	-10.41	-0.7
β-Alanine	-12.63	-3.0
β-Aminobutyric acid	-12.52	-3.1
β-Aminoisobutyric acid	-12.53	-2.9
β-Lactic acid	-13.90	-1.0
γ-Aminobutyric acid	-12.44	-3.2

γ-Hydroxybutyric acid	-13.91	-0.6
δ-Aminopentanoic acid	-12.44	-2.6
δ-Hydroxyvaleric acid	-13.83	-0.4
ε-Aminohexanoic acid	-12.39	-3.0
ε-Hydroxycaproic acid	-13.79	-0.4
Methane	-16.61	0.6
Ethane	-15.16	1.3
Propane	-14.74	1.8
Isobutane	-14.48	2.1

**Secondary metabolites**	**HLG [eV]**	**XLogP**
(-)-Acanthocarpan	-8.95	1.5
(-)-Epiafzelechin	-10.37	0.7
(-)-γ-Cadinene	-10.84	4.3
(-)-Hygrine	-11.42	0.5
(-)-Hygroline	-11.46	0.9
(-)-Nissolin	-9.90	2.3
(-)-Sativan	-10.11	3.3
(-)-Vestitol	-10.18	2.9
(-)-Vestitone	-10.20	2.3
(+)-Abscisic acid	-10.23	1.6
(+)-α-Pinene	-10.74	2.8
(+)-β-Caryophyllene	-10.75	4.4
(+)-Camphor	-12.30	2.2
(+)-Catechin	-10.21	0.4
(+)-Cubenene	-10.61	4.5
(+)-Dalbergioidin	-10.48	2.2
(+)-Elaeocarpine	-10.99	2.6
(+)-Gallocatechin	-10.27	0.0
(+)-Germacrene A	-10.50	4.7
(+)-Germacrene D	-9.12	4.7
(+)-Maackiain	-8.93	2.5
(+)-Medicarpin	-9.97	2.6
(+)-Neomatatabiol	-12.96	2.3
(+)-Pimaric acid	-10.86	5.5
(+)-Pinoresinol	-9.93	2.3
(+)-Prosopinine	-11.44	3.6
(+)-Sandaracopimaradiene	-10.61	7.0
(+)-Sophorol	-9.15	2.2
(5Z,8Z,11Z,14Z,17Z)-Icosapentaenoic acid	-10.66	5.6
(6S)-Hydroxyhyoscyamine	-10.99	0.9
6-Gingerol	-10.17	2.5
6-Shogaol	-10.07	3.7
8-Gingerol	-10.17	4.2
13-(2-Methylcrotonoyl)oxylupanine	-10.64	2.4
13-Hydroxylupanine	-11.03	0.6
1-O-Sinapoyl-β-D-glucose	-9.71	-0.3
2',6'-Dihydroxy-4'-methoxydihydrochalcone	-8.96	1.7
2,7,11-Cembratrien-4,6-diol	-10.60	4.0
24-Methylenecycloartanol	-10.95	10.3
2-Ketoepimanool	-11.14	3.8
3-Hydroxystachydrine	-10.51	0.6
4-Coumaroylshikimate	-9.97	0.6
4-Coumaryl alcohol	-9.61	1.3
4-Hydroxycinnamyl aldehyde	-9.97	1.8
5,6-Dehydrokawain	-8.35	2.8
5,6-Dehydrolupanine	-9.92	1.3
5-Deoxyleucocyanidin	-10.21	0.7
5-Deoxyleucopelargonidin	-10.60	1.0
5-Hydroxyconiferaldehyde	-9.84	1.1
5-Hydroxyferulate	-9.90	1.8
6-Paradol	-10.13	3.8
7,3'-Dihydroxy-4'-methoxy-8-methylflavan	-10.05	3.4
7,4'-Dihydroxyflavan	-10.25	3.1

7-Hydroxyflavan	-10.30	3.5
8-(1,1-DMA)kaempferide	-9.66	4.8
Abietate	-9.30	4.8
Abrusoside A	-11.54	5.3
Acetylintermedine	-10.95	0.1
Acetyllycopsamine	-10.98	0.1
Acetylpseudotropine	-11.37	1.3
Acetyltropine	-11.67	1.3
Achillin	-11.32	1.6
Adenocarpine	-9.22	2.4
Afzelechin	-10.52	0.7
Agrostophyllin	-9.09	3.6
Albafuran A	-8.87	6.7
Albine	-9.54	1.3
Alexine	-11.01	-1.7
Alisol A	-10.37	3.8
Alisol B	-9.89	4.8
Alisol C	-10.84	3.9
Alizarin	-9.57	3.2
Aloe emodin anthrone	-10.25	2.3
Aloin A	-9.85	-0.1
Aloin B	-10.34	-0.1
α-Amyrin	-10.44	9.0
α-Cedrol	-12.80	3.9
α-Eudesmol	-10.58	3.5
α-Ionone	-11.03	3.0
α-Irone	-10.93	3.2
α-Kosin	-10.49	5.6
Amabiline	-11.01	0.5
Ambrosin	-11.73	2.6
Amentoflavone	-9.34	5.0
Ammodendrine	-9.81	0.3
Ammothamnine	-10.93	1.0
Anacrotine	-11.04	0.1
Anagyrine	-9.70	1.6
Anatoxin a	-8.65	0.8
Andrographolide	-10.97	2.2
Androstenedione	-11.33	2.7
Anethole	-9.49	3.3
Angelicin	-10.09	2.0
Angularine	-11.26	0.6
Angustifoline	-10.90	1.4
Anibine	-9.20	1.0
Anisodamine	-11.01	0.9
Aphidicol-15-ene	-10.43	6.7
Aphidicol-16-ene	-9.31	6.9
Aphidicolan-16β-ol	-9.07	5.8
Aphidicolin	-12.20	2.5
Aphylline	-11.24	1.7
Apiforol	-10.26	1.6
Apigeninidin	-8.91	3.0
Apoatropine	-10.20	3.6
Apohyoscine	-10.18	2.6
Arctigenin	-9.90	3.6
Arctiin	-10.07	1.8
Argentine	-9.35	0.5
Argyrolobine	-10.08	0.9
Asebogenin	-10.04	3.0
Asebotoxin II	-11.06	1.4
Aspidinol	-10.53	2.6
Astrocasine	-8.13	3.2
Astrophylline	-10.06	3.0
Athyriol	-10.32	2.4
Atropine	-11.06	1.8
Aurasperone D	-8.67	6.0
Aureusidin	-8.92	2.7
Auriculine	-10.33	4.4

Australine	-11.26	-1.7
Baptifoline	-9.62	0.6
Batatasin I	-8.86	4.0
Batatasin IV	-10.11	3.4
Belladonnine	-10.73	6.0
Bellendine	-11.14	1.0
Benzoylecgonine	-11.37	-0.3
Bergaptol	-9.65	2.0
β-Amyrin	-10.36	9.2
β-Cadinene	-10.71	4.0
β-Ionone	-9.87	2.9
Betulin	-10.57	8.3
Betulinic acid	-10.94	8.2
Bikaverin	-8.72	3.6
Blestriarene B	-8.60	6.5
Borneol	-12.82	2.7
Boschnialactone	-13.13	2.0
Bracteatin	-8.92	2.4
Broussin	-10.26	3.4
Brugine	-9.91	2.2
Buchananine	-11.63	-2.0
Buddledin A	-10.87	3.5
Butein	-9.38	2.8
Caffeic acid	-9.85	1.2
Caffeic aldehyde	-9.74	1.1
Caffeyl alcohol	-9.41	1.1
Calanolide A	-9.80	3.8
Calpurnine	-9.92	2.0
Calystegin A3	-12.04	-1.5
Camelliol C	-10.43	9.7
Camoensine	-9.57	1.2
Canaliculatol	-9.39	6.8
Cannabichromene	-9.58	6.9
Cannabidiol	-10.27	6.5
Cannabidivarin	-10.27	5.4
Cannabinol	-9.66	6.1
Carnosol	-10.37	4.4
Carolinianine	-10.76	1.1
Carpaine	-11.35	6.3
Casbene	-10.00	5.4
Cassine	-11.48	4.1
Castanospermine	-11.51	-2.2
Caulophylline	-9.65	0.7
Cembrene	-9.46	6.0
Cernuine	-10.98	2.8
Chalepensin	-9.91	4.3
Chelidonic acid	-10.87	-0.4
Chlorogenate	-9.84	-0.4
Chlorophorin	-8.64	6.6
Cholesterol	-10.43	8.7
Chrysanthemic acid	-10.86	3.4
Chrysarobin	-10.41	3.6
Cichorine	-10.47	0.8
Cimifugin	-10.35	0.6
Cimigenol	-10.84	5.2
Cinegalline	-10.95	2.1
Cinerin I	-10.52	5.0
Cinerin II	-11.11	4.0
Cinnamoylcocaine	-10.10	2.7
cis-3,4-Leucopelargonidin	-10.54	0.7
cis-Hinokiresinol	-9.49	4.4
Clivoline	-10.09	2.0
Cnicin	-10.37	0.2
Cocaine	-11.35	2.3
Cochlearine	-10.79	2.6
Coelogin	-9.18	2.6
Confertin	-11.83	2.1

Coniferin	-9.55	-1.3
Coniferyl aldehyde	-9.77	1.5
Coniferyl ferulate	-9.20	3.6
Coniin	-11.71	2.0
Convolamine	-10.55	3.1
Convolvine	-10.64	2.7
Copalyl diphosphate	-10.77	3.9
Coriamyrtin	-11.62	0.5
Coumarin	-10.51	1.4
Coumestrol	-9.22	2.8
Crataegolic acid	-10.59	6.5
Crotanecine	-10.80	-2.3
Cryptophorine	-8.31	3.8
Cucurbitacin D	-10.77	2.1
Curcumin	-9.48	3.2
Cuscohygrine	-11.23	1.0
Cycloartenol	-10.60	9.8
Cytisine	-9.73	0.2
D-(-)-Anaferine	-11.33	0.8
Daidzein	-9.59	2.5
Darlingine	-10.97	1.4
Dasytrichone	-9.58	2.9
Datiscetin	-9.82	1.9
Davidigenin	-10.08	3.0
Dehydroabietic acid	-10.51	5.6
Dehydrodieugenol	-9.55	4.9
Δ9-Tetrahydrocannabinol	-10.14	7.0
Dendrolasin	-10.14	5.1
Deoxymannojirimycin	-11.99	-2.3
Deoxynupharidine	-10.24	3.5
Diferulic acid	-9.77	2.7
Dihydrokaempferol	-10.66	1.8
Dihydromethysticin	-9.21	2.6
Dihydroresveratrol	-10.31	3.1
Dimethamine	-8.23	0.3
Dioscorine	-11.14	1.5
Dipterocarpol	-10.67	8.2
Doronine	-11.52	1.5
Dracorubin	-7.25	5.3
Ecgonine	-11.32	-1.8
Ecgonine methyl ester	-11.50	0.6
Echimidine	-10.52	-0.1
Elaeokanine C	-11.09	1.4
Elemicin	-10.77	2.5
ent-Kaurene	-11.00	6.9
ε-Viniferin	-8.80	5.4
Equol	-10.22	3.0
Eriodictyol	-10.23	2.0
Estrone	-10.33	3.1
Eucommin A	-10.05	0.4
Eugenol	-10.06	2.0
Euphol	-10.15	8.9
Europine	-11.21	-1.3
Euscaphic acid	-10.32	5.0
Euxanthone	-10.21	2.8
Fagomine	-11.95	-1.4
Farnesol	-10.54	4.8
Farnesyl diphosphate	-10.27	2.6
Farrerol	-10.39	3.1
Fernene	-10.55	10.5
Ferreirin	-10.44	2.5
Ferulic acid	-9.92	1.5
Fisetinidol	-10.15	0.7
Flavidin	-9.06	2.7
Fraxetin	-9.87	1.2
Friedelin	-10.55	9.8
Fucosterol	-10.39	8.9

Fulvine	-10.93	0.2
Fusaric acid	-11.60	2.6
Fustin	-10.25	1.3
Futoquinol	-8.92	3.9
Galanolactone	-11.34	4.6
Gambiriin C	-9.83	2.7
γ-Bisabolene	-10.45	4.7
Garbanazol	-10.50	1.6
Gartanin	-9.73	5.9
Geigerin	-11.77	0.8
Genipin	-11.00	-0.7
Genkwanin	-9.85	2.1
Gentiopicrin	-10.43	-1.2
Geranial	-10.89	3.0
Geraniol	-10.75	2.9
Geranyl diphosphate	-10.48	0.7
Geranylgeraniol	-10.43	6.6
Geranylgeranyl diphosphate	-10.17	4.4
Gibberellic acid	-11.33	0.2
Gibberellin A1	-11.32	0.2
Gibberellin A24	-10.93	2.3
Gibberellin A4	-11.00	1.7
Gibberellin A7	-10.99	1.7
Ginkgolide A	-13.12	0.6
Ginkgolide B	-10.36	-0.4
Ginkgolide J	-13.09	-0.4
Girgensonine	-10.78	2.2
Glepidotin C	-10.18	4.3
Gnetin A	-8.72	3.9
Gossypetin	-9.26	1.8
Gossypol	-8.53	6.9
Grandinol	-10.93	2.7
Granilin	-11.41	0.9
Grayanotoxin I	-12.13	0.8
Guaiol	-10.35	3.1
Guibourtinidol-(4α-6)-catechin	-9.83	3.1
Gypsogenin	-10.24	6.7
Hamaudol	-10.43	2.1
Harzianopyridone	-9.98	1.7
Helenalin	-11.66	1.4
Heliangin	-11.22	1.9
Heliosupine	-10.88	-0.1
Heliotridine	-10.75	-1.3
Heliotrine	-11.14	0.1
Hesperetin	-10.20	2.4
Hinokiflavone	-9.77	4.4
Hispidol	-9.01	2.9
Hopane-29-acetate	-11.98	10.8
Humulene	-10.19	4.5
Hydrangenol	-10.71	3.2
Hygrine	-11.34	0.5
Hypericin	-6.77	5.7
Ichangin	-9.93	0.8
Indicine	-11.13	-0.4
Inflexin	-11.78	1.8
Ingenol	-10.17	0.2
Integerrimine	-10.99	1.1
Intermedine	-10.99	-0.4
Iridodial	-12.89	2.6
Iridomyrmecin	-13.08	2.6
Irisolidone	-9.28	3.0
Isobatatasin I	-8.94	4.0
Isocaryophyllene	-10.68	4.4
Isochamaejasmin	-10.40	4.6
Isochlorogenic acid b	-9.81	1.5
Isoliquiritigenin	-9.59	3.2
Isolobinine	-10.98	3.0

Isolycopsamine	-10.87	-0.4
Ivalin	-11.30	2.0
Jacareubin	-9.57	3.4
Jacobine	-11.08	0.3
Jatrophatrione	-9.81	3.0
Jatrophone	-9.49	3.7
Justicidin A	-8.71	4.0
Juvabione	-11.41	3.5
Juvenile hormone III	-10.83	4.0
Kaurenoic acid	-11.04	5.4
Kawain	-9.90	2.5
Kazinol A	-9.85	6.6
Khellin	-9.20	2.3
Kievitone	-10.24	4.1
Knightinol	-11.01	2.1
Knipholone	-8.29	4.2
Kuwanone G	-9.26	7.3
L-(+)-Anaferine	-11.33	0.8
Lactucin	-11.28	-0.7
Lamprolobine	-10.99	1.2
Lasiocarpine	-10.83	0.5
Lathyrol	-10.82	2.2
Latifoline	-10.68	0.9
Ledol	-11.94	3.7
Lentiginosine	-11.10	-0.3
Leptosidin	-8.94	2.5
Leucocyanidin	-10.19	-0.8
Leucodelphinidin	-10.10	0.0
Levopimaric acid	-9.17	4.8
L-Hyoscyamine	-11.08	1.8
Libanorin	-9.77	3.9
Limonene	-10.91	3.4
Limonin	-10.20	1.8
Littorine	-10.88	2.7
Lobelanidine	-10.53	3.6
Lobelanine	-11.08	3.9
Lobelin	-11.08	3.8
Lonchocarpenin	-8.83	5.7
Lonchocarpol A	-10.00	6.2
Loroglossol	-9.15	3.4
Lotisoflavan	-9.87	2.9
L-Pipecolate	-12.02	-2.3
Lunularic acid	-10.26	3.5
Lunularin	-10.36	3.5
Lupan-3β,20-diol	-11.89	8.4
Lupanine	-11.12	1.6
Lupeol	-10.85	9.9
Lupeol acetate	-10.88	10.4
Lupinine	-11.02	1.2
Lupulone	-9.52	6.7
Luteoforol	-10.19	1.3
Luteolin	-9.52	1.4
Luteolinidin	-8.68	4.0
Luteone	-9.37	4.2
Lycocernuine	-10.98	1.8
Lycopsamine	-11.00	-0.4
Macrophylline	-11.33	1.3
Maltol	-10.99	0.4
Malvidin	-8.52	4.0
Marchantin A	-10.05	6.4
Maritimetin	-8.77	2.2
Marmesin	-9.73	1.9
Matrine	-11.09	1.6
Mearsine	-12.36	0.6
Meconic acid	-10.49	0.0
Meteloidine	-11.29	0.6
Methysticin	-9.03	2.4

Micromelin	-10.36	0.3
Mikanolide	-12.19	0.2
Millettone	-9.31	3.6
Miroestrol	-10.16	0.2
Monocrotaline	-11.27	-0.7
Moracin A	-8.67	3.2
Mucronulatol	-10.24	2.9
Mukaadial	-11.97	1.7
Mulberrofuran A	-8.62	7.0
Myrcene	-10.95	4.3
Myristicin	-9.34	2.9
Nagilactone C	-10.09	-0.2
Nemorensine	-11.38	1.7
Neoabietadiene	-9.04	6.6
Neocembrene	-10.45	5.9
Neral	-10.95	3.0
Nigrifactin	-8.61	2.5
Nitramine	-11.07	1.7
N-Methylpelletierine	-11.26	0.8
Nomilin	-10.12	2.6
Norhyoscyamine	-10.98	1.4
Nuttalline	-11.01	0.6
O-7-Angelylheliotridine	-10.65	0.5
Obacunone	-10.15	3.2
Obtusin	-9.43	3.0
Odoratol	-10.54	3.1
Okanin	-9.31	2.5
Oleanolic acid	-10.33	7.5
Orchinol	-8.82	3.4
Orobol	-9.26	2.3
Oryzalexin A	-11.12	4.6
Otonecine	-11.68	-0.4
Oxyresveratrol	-8.75	2.8
Pachyrrhizone	-9.07	3.1
Parsonsine	-11.01	1.0
Patchoulol	-12.25	4.1
Pelletierine	-11.67	0.4
Pentalenene	-10.67	4.7
Petasitenine	-11.77	0.6
Phalaenopsine T	-10.89	2.1
Phaseic acid	-10.29	0.5
Phillyrin	-9.77	0.0
Phloretin	-10.12	2.6
Phorbol	-10.98	-0.8
Phyllalbine	-10.52	3.0
Phyllodulcin	-10.25	3.1
Physcion	-9.44	3.0
Physoperuvine	-11.69	1.0
Phytol	-11.10	8.2
Phytuberin	-10.20	2.3
Phytyl diphosphate	-10.95	6.0
Piceatannol	-8.77	2.9
Picolinic acid	-11.90	0.8
Pimaradiene	-10.79	7.0
Pinobanksin	-10.84	2.2
Pinocembrin	-10.81	2.7
Pinocembrin chalcone	-9.92	3.2
Pinosylvin	-9.16	3.5
Piperideine	-12.24	0.1
Piperine	-8.64	3.5
Piplartine	-9.93	2.1
Pisatin	-9.03	1.7
Platyphylline	-11.06	1.9
Pleniradin	-9.70	0.7
Podocarpic acid	-10.16	4.1
Podophyllotoxin	-9.69	2.0
Pomiferin	-9.32	5.5

Poststerone	-11.63	0.3
Proanthocyanidin A2	-9.66	2.4
Procyanidin B4	-9.66	2.4
Propapyriogenin A2	-10.82	4.4
Prostratin	-10.79	0.7
Pseudoconhydrine	-11.82	1.0
Pseudoecgonine	-11.18	-1.8
Pseudopelletierine	-11.60	0.7
Pseudotropine	-11.25	0.8
Psoralidin	-9.13	4.7
Ptaeroglycol	-9.96	0.9
Purpurin	-9.22	2.9
Pyrethrin I	-9.53	5.4
Pyrethrin II	-9.69	4.4
Quercetin	-9.33	1.5
Quillaic acid	-10.16	6.1
Quinquangulin	-9.04	3.3
Radiatin	-11.40	2.1
Randainol	-9.36	3.7
Resiniferonol	-11.00	-0.5
Resveratrol	-8.92	3.1
Retamine	-10.71	1.6
Retronecine	-10.71	-1.3
Retrorsine	-10.83	0.6
Rhombifoline	-9.67	1.8
Riddelline	-10.87	0.2
Rinderine	-11.16	-0.4
Robustaflavone	-9.68	5.0
Rosmarinine	-11.12	1.0
Rotenone	-9.83	4.1
Rottlerin	-9.52	5.9
Royleanone	-8.61	4.4
Rubrofusarin	-9.20	3.0
Rugosal	-11.73	1.7
Rutaevin	-10.09	1.3
Safranal	-9.81	2.1
Sainfuran	-8.86	3.2
Santin	-9.47	3.1
Sarracine	-10.56	1.8
Sciadopitysin	-9.54	6.0
Sclareol	-11.76	4.9
Scopolamine	-11.11	0.9
Scopoline	-9.56	-0.2
Secologanate	-11.07	-1.9
Secologanin	-11.31	-1.6
Sedamin	-11.04	2.4
Senecionine	-11.03	1.1
Senecionine N-oxide	-11.08	0.5
Seneciphylline	-11.09	0.7
Senecivernine	-10.88	1.2
Senkirkine	-11.19	1.4
Sequirin A	-10.32	2.2
Sesquicarene	-10.46	4.8
Shikodonin	-11.61	1.4
Simplexoside	-9.30	0.7
Sinapate	-10.00	1.5
Sinapoyl aldehyde	-9.86	1.4
Slaframine	-11.52	0.0
Sojagol	-8.97	4.1
Solenopsin A	-11.52	6.7
Sophoramine	-9.55	1.6
Sorgolactone	-10.83	2.2
Soularubinone	-11.20	-0.5
Sparteine	-10.82	2.5
Stachydrine	-10.17	1.0
Steganacin	-9.30	2.9
Steviol	-11.08	3.8

Strobamine	-9.73	2.0
Supinidine	-10.75	-0.3
Supinine	-11.12	0.5
Surinamensin	-9.30	4.1
Swainsonine	-11.47	-1.3
Swerchirin	-9.93	2.7
Swertiamarin	-11.08	-2.0
Symlandine	-10.91	1.3
Symphytine	-10.87	1.3
Syringin	-9.83	-1.3
Taraxerol	-10.52	9.3
Taxifolin	-10.26	1.5
Taxodone	-9.08	4.1
Tenulin	-12.02	0.3
Tephrosin	-9.87	3.0
Tephrowatsin A	-9.73	4.6
Testosterone	-11.29	3.3
Tetrahymanol	-12.02	10.1
Thermospine	-9.62	1.6
Thymol	-10.38	3.3
Tigloidine	-11.40	2.5
Tinctorine	-9.47	1.8
Toxicarol	-9.71	3.9
Trachelanthamidine	-11.43	0.5
trans-trans-cis-Geranylgeranyl diphosphate	-10.44	4.4
Triangularine	-10.68	1.0
Tricin	-9.87	1.7
Tropacocaine	-11.13	3.0
Tropine	-11.25	0.8
Tropinone	-11.71	0.3
Tussilagine	-11.46	0.6
Tutin	-11.52	-0.5
Umbelliferone	-10.09	1.6
Uplandicine	-11.25	-1.3
Ursolic acid	-10.40	7.3
Usaramine	-10.81	0.6
Valeroidine	-11.17	1.6
Vernoflexin	-10.96	3.3
Vignafuran	-8.33	3.6
Visnagin	-9.61	2.3
Vulgarin	-12.26	1.2
Wedelolactone	-9.43	2.4
Wogonin	-9.91	3.0
Xanthohumol	-9.56	5.1
Yakuchinone A	-10.04	3.6
Yangonin	-8.32	2.7
Zapoterin	-10.08	2.0
Zingerone	-10.12	0.8
